# Prenatal antibiotics exposure and the risk of autism spectrum disorders: A population-based cohort study

**DOI:** 10.1371/journal.pone.0221921

**Published:** 2019-08-29

**Authors:** Amani F. Hamad, Silvia Alessi-Severini, Salaheddin M. Mahmud, Marni Brownell, I fan Kuo

**Affiliations:** 1 College of Pharmacy, Rady Faculty of Health Sciences, University of Manitoba, Winnipeg, Canada; 2 Manitoba Centre for Health Policy, Max Ray College of Medicine, Rady Faculty of Health Sciences, University of Manitoba, Winnipeg, Canada; 3 Department of Community Health Sciences, Max Ray College of Medicine, Rady Faculty of Health Sciences, University of Manitoba, Winnipeg, Canada; 4 Vaccine and Drug Evaluation Centre, University of Manitoba, Winnipeg, Canada; University of Missouri Columbia, UNITED STATES

## Abstract

**Background:**

Prenatal antibiotic exposure induces changes in infants’ gut microbiota composition and is suggested as a possible contributor in the development of autism spectrum disorders (ASD). In this study, we examined the association between prenatal antibiotic exposure and the risk of ASD.

**Methods:**

This was a population-based cohort study utilizing the Manitoba Population Research Data Repository. The cohort included 214 834 children born in Manitoba, Canada between April 1, 1998 and March 31, 2016. Exposure was defined as having filled one or more antibiotic prescription during pregnancy. The outcome was autism spectrum disorder diagnosis. Multivariable Cox proportional hazards regression was used to estimate the risk of developing ASD in the overall cohort and in a sibling cohort.

**Results:**

Of all subjects, 80 750 (37.6%) were exposed to antibiotics prenatally. During follow-up, 2965 children received an ASD diagnosis. Compared to children who were not exposed to antibiotics prenatally, those who were exposed had a higher risk of ASD: (adjusted HR 1.10 [95% CI 1.01, 1.19]). The association was observed in those exposed to antibiotics in the second or third trimester (HR 1.11 [95% CI 1.01, 1.23] and 1.17 [95% CI 1.06, 1.30], respectively). In the siblings’ cohort, ASD risk estimate remained unchanged (adjusted HR 1.08 [95% CI 0.90, 1.30], although it was not statistically significant.

**Conclusions:**

Prenatal antibiotic exposure is associated with a small increase in the risk of ASD. Given the potential of residual confounding beyond what it was controlled through our study design and because of possible confounding by indication, such a small risk increase in the population is not expected to be clinically significant.

## Introduction

Autism spectrum disorders (ASD) are characterized by impairment in social communication and interaction with repetitive patterns of behavior [1]. The burden of ASD is significant with 62 million cases worldwide [2]. Genetics are primary contributors to the development of ASD; however, the increasing prevalence of ASD suggests a role of environmental factors [3–7].

Abnormal composition of microbiota, the community of microorganisms residing in the human body, has been observed in children with ASD and is proposed as a contributor to ASD development [8–11]. Recent research have shown that the fetal gut is not germ free and suggests maternal microbiota transfer before birth [12, 13]. Antibiotic-altered microbiota administered to pregnant mice demonstrated transmission of the same alteration of microorganisms to the offspring. Despite absence of direct exposure to antibiotics, the offspring maintained this microbial composition for at least 21 weeks and were found to be at higher risk of developing colitis [14]. In another study, treating pregnant mice with antibiotics directly resulted in persistent reduction in offspring gut microbiota diversity and in immunological alterations [15]. Antibiotic-induced changes in fetal microbiota composition can disrupt the gut-brain axis, potentially impairing neurodevelopment and increasing the risk of ASD [8, 9, 16].

Although previous observational studies reported several prenatal and postnatal environmental factors as predictors of ASD [17–19], potential of confounding and other study flaws limited the clinical applicability of these associations. In this study, we aimed to examine the association between prenatal antibiotic exposure and the risk of ASD. Antibiotics over-prescription and inappropriate use are often observed during pregnancy [20–22]. Given the high frequency of antibiotic prescribing in this population [20, 23], identifying an association between antibiotic use and ASD, if any, would be of public health interest.

## Methods

### Design and subjects

This was a population-based cohort study utilizing administrative health data from the Manitoba Population Research Data Repository housed at the Manitoba Centre for Health Policy. The Repository is a collection of administrative, registry, survey and other data that come from different provincial government departments such as health, education and justice. Under a universal, provincial health delivery system, the Repository captures all encounters by all residents with the healthcare system including physician visits and drug dispensation, collected for claim purposes. Patient records in the Repository are de-identified. Scrambled Personal Health Identification Numbers (PHIN) are used for linkage among different databases.

The cohort consisted of all live births identified in the Manitoba Health Insurance Registry between April 1, 1998 and March 31, 2016. Children were required to have continuous enrollment with Manitoba Health for at least 18 months after their birthdate, which was also the cohort index date, to ensure that they have met the minimum age of ASD diagnosis [24]. In addition, to obtain data on maternal covariates, the mothers were required to have at least two years of Manitoba Health enrollment prior to index date. Children were followed until a diagnosis of ASD, migration out of province, 18th birthday, death or end of study period (March 31, 2016), whichever occurred first. Subjects with missing data on any of the relevant covariates were excluded from the cohort. To account for unmeasured familial environmental and genetic confounders that are shared between siblings, we identified a cohort of maternal siblings who have different exposure status to prenatal antibiotics, i.e., one or more of the siblings were exposed to antibiotics prenatally and one or more were not.

Other data sources of the study included the Drug Program Information Network (DPIN), In-hospital Pharmaceuticals, Hospital Abstracts, physician claims from the Medical Services database, the Manitoba Education and Training Special Needs Funding data, the Hospital Newborn to Mother Link Registry, BabyFirst—Families First Screen and the Social Allowances Management Information Network (SAMIN) (S1 Table). The study was approved by the University of Manitoba Health Research Ethics Board and the Health Information Privacy Committee of Manitoba Health, Seniors and Active Living.

### Exposure

The exposure was identified in the Drug Program Information Network (DPIN), a record of outpatient drug dispensations, and was defined as having filled one or more antibiotic prescriptions during pregnancy (S2 Table). The first day of pregnancy was estimated as the difference between birth date and gestational age. Gestational age is approximated from the first date of women’s last menstrual period and is identified in the Hospital Newborn to Mother Link Registry. In secondary analyses, the exposure was examined based on the pregnancy trimester, number of antibiotic courses received, cumulative duration and class of antibiotic. Grouping of these variables was based on their frequency in the cohort.

### Outcome

The primary outcome was ASD diagnosis after 18 months of age and included childhood autism, atypical autism, Asperger’s disorder, childhood disintegrative disorder, other pervasive developmental disorders and pervasive developmental disorders not otherwise specified [1].

The 9^th^ and 10^th^ revisions of the International Classification of Disease (ICD) coding system were used to identify ASD diagnosis. ASD was defined as one or more hospitalization with an ASD code (ICD-9 299.0, 299.1, 299.8 or 299.9, or ICD-10 F84.0, F84.1, F84.3, F84.5, F84.8 or F84.9), one or more physician visit with ASD code (ICD-9 code of 299) or presence of an "ASD" identifier in the Manitoba Education and Training Special Needs Funding data [7, 25, 26]. A validation study reported a positive predictive value of 88% using one or more hospitalizations or physician visits [27]. Including educational data as a source to identify ASD is expected to increase the sensitivity [28].

### Covariates

The models were adjusted for region of residence (urban or rural), socioeconomic status (SES), mothers’ age at delivery (less than 30, 30 to 39, and 40 years or greater), prenatal use of medications and maternal medical conditions of interest (S3 and S4 Tables). SES was measured using the Socio-Economic Factor Index (SEFI), an area level measure derived from Census data. In addition, receiving income assistance was explored as a proxy of individual level SES. Data on prenatal smoking, alcohol or drug use were obtained from the BabyFirst—Families First Screen. A large amount of missing data was observed for these variables; accordingly, they were not included in the main analysis but were explored in a sensitivity analysis restricted to children who had data on these variables. Number of mothers’ physician visits in the year prior to pregnancy was included as a measure of healthcare access. Indication of the dispensed antibiotic is not reliably captured in the administrative databases; hence, we were not able to account for the specific indication for which the antibiotics were prescribed. The models were also adjusted for child covariates including sex, size for gestational age, mode of delivery, birth complications, breast feeding initiation, multiple births, birth order (first born or subsequent), season of birth, year of birth, early childhood antibiotics use and medical conditions of interest (S4 Table).

### Statistical analysis

Multivariable Cox proportional hazards regression was used to examine the association between antibiotic exposure and ASD diagnosis. To account for correlation among siblings, regression models were stratified by the mothers to examine this association within the sibling cohort. The analysis was stratified by sex and region to examine potential effect modification. Multicollinearity among covariates and interactions with antibiotic exposure were explored. Proportional hazards assumption was tested by examining the correlation between follow-up time and Schoenfeld residuals of the independent variables.

In the planned sensitivity analyses, we restricted the cohort to children whose mothers had an ICD code for infection during pregnancy. We applied a stricter ASD identification algorithm, which required one hospitalization or two physician claims within three years or one physician claim plus educational special needs funding for ASD within three years. We varied the minimum age for ASD diagnosis to one and two years old, we included inpatient antibiotic use in identifying the exposure and we included prenatal smoking, alcohol or drug use. In addition, we conducted two negative-control analyses by examining maternal antibiotic exposure in the year before pregnancy and the year after birth. The statistical software SAS^®^ 9.4 (SAS Institute; Cary, NC) was used for all data analyses.

## Results

### Description of study population

**Fig 1:**
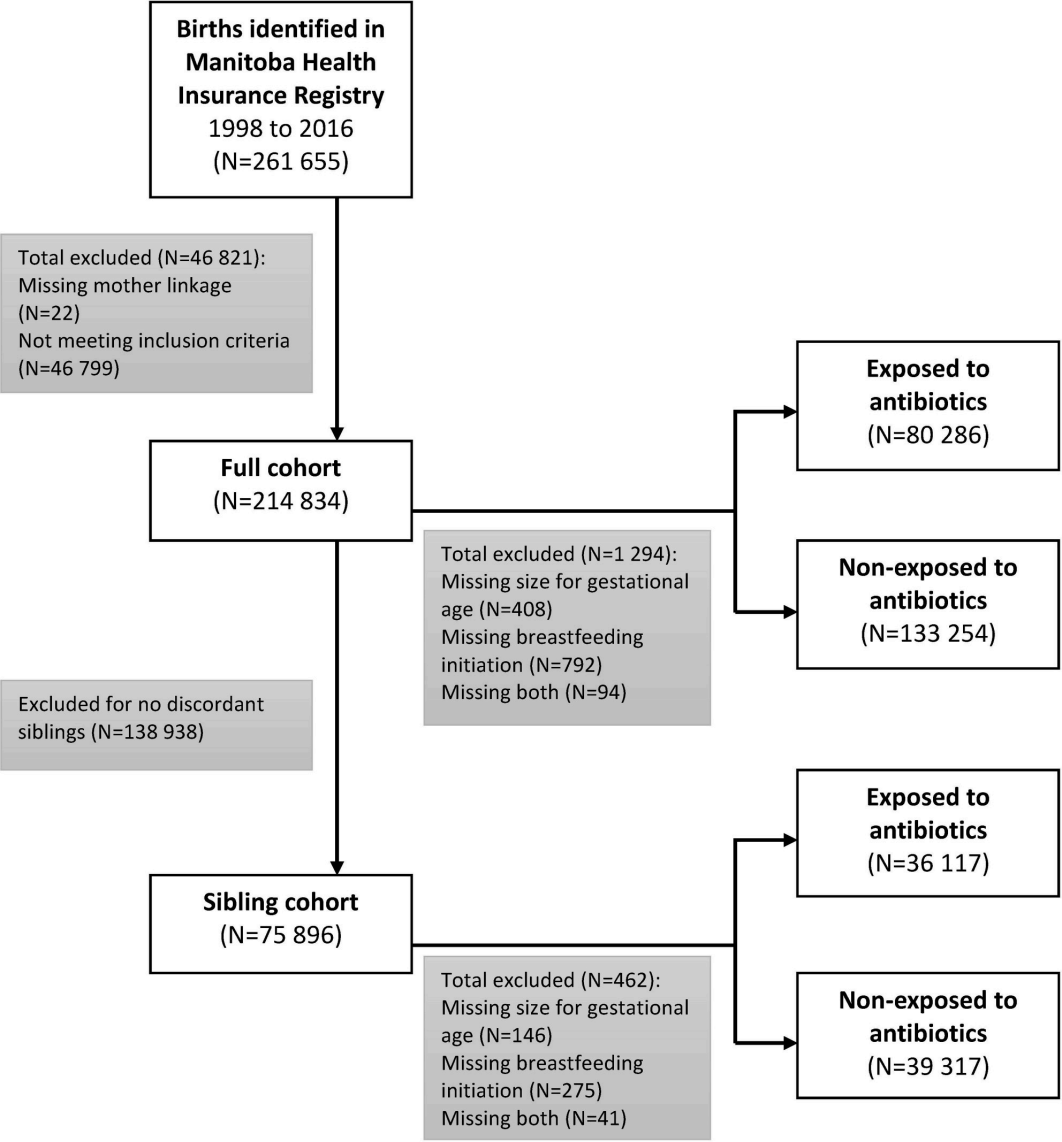
Study populations: Overall and sibling cohort.

A total of 214 834 children met the inclusion criteria (Fig 1). About 51% were males and 54.4% resided in an urban region (Table 1); 37.6% of the children were exposed to antibiotics prenatally. The majority were exposed to one antibiotic course (62.8%) or were exposed for less than or equal to two weeks (74.1%). 54.6% were exposed to a penicillin antibiotic (S5 Table).

**Table 1:** Characteristics of study cohort: Overall and by antibiotic exposure status.

Characteristic: No (%) ^a^	All subjects N = 214 834	Antibiotic use during pregnancy	
		NO N = 134 084	YES N = 80 750
**Male**	110 107 (51.3)	68 691 (51.2)	41 416 (51.3)
**Urban region**	116 865 (54.4)	74 887 (55.9)	41 978 (52.0)
**Socioeconomic status (SES)**^**b**^:			
**High** **Middle** **Low-mid** **Low**	21 212 (9.9) 77 708 (36.2) 67 059 (31.2) 48 855 (22.7)	14 912 (11.1) 50 861 (37.9) 40 410 (30.1) 27 901 (20.8)	6 300 (7.8) 26 847 (33.3) 26 649 (33.0) 20 954 (26.0)
**Receipt of income assistance** ^**c**^	37 158 (17.3)	17 058 (12.7)	20 100 (24.9)
**Mothers age at delivery:**			
**< 30** **30–39** **> = 40**	128 229 (59.7) 81 963 (38.2) 4 642 (2.2)	76 411 (57.0) 54 602 (40.7) 3 071 (2.3)	51 818 (64.2) 27 361 (33.9) 1 571 (2.0)
**Breastfeeding initiation** ^**d**^	172 952 (80.8)	110 425 (82.7)	62 527 (77.7)
**Multiple birth** ^**e**^	5 383 (2.5)	3 246 (2.4)	2 137 (2.7)
**Caesarian section delivery**	43 782 (20.4)	26 810 (20.0)	16 972 (21.0)
**Birth complications**	21 022 (9.8)	13 092 (9.8)	7 930 (9.8)
**First born child**	80 758 (37.6)	52 334 (39.0)	28 424 (35.2)
**Small for gestational age** ^**f**^	16 507 (7.7)	10 445 (7.8)	6 062 (7.5)
**Prenatal alcohol/drug use** ^**g**^	12 959 (12.3)	7 265 (10.9)	5 694 (14.7)
**Prenatal smoking** ^**h**^	20 844 (19.4)	10 687 (15.8)	10 157 (25.7)
**Childhood medical conditions:**			
** Infections:**			
**None** **Mild-moderate** ^**i**^ **Severe** ^**j**^ **Epilepsy** **Neonatal jaundice** **Other developmental disabilities** **Asthma**	67 430 (31.4) 134 794 (62.7) 12 610 (5.9) 1 056 (0.5) 20 517 (9.6) 859 (0.4) 26 641 (12.4)	48 169 (35.9) 78 881 (58.8) 7 034 (5.3) 589 (0.4) 11 934 (8.9) 489 (0.4) 13 522 (10.1)	19 261 (23.9) 55 913 (69.2) 5 576 (6.9) 467 (0.6) 8 583 (10.6) 370 (0.5) 13 119 (16.3)
**Early life antibiotics exposure** ^**k**^	94 024 (43.8)	51 524 (38.4)	42 500 (52.6)
**Maternal medical conditions:**			
**Mood and anxiety disorders** **Schizophrenia** **Diabetes** **Infections**	15 885 (7.4) 196 (0.1) 6 085 (2.8) 67 559 (31.5)	7 493 (5.6) 87 (0.1) 3 268 (2.4) 22 214 (16.6)	8 392 (10.4) 109 (0.1) 2 817 (3.5) 45 345 (56.2)
**Prenatal medications use:**			
**Antidepressants** **Antipsychotics** **Anticonvulsants** **Cardiovascular medications**	5 759 (2.7) 613 (0.3) 1 282 (0.6) 3 370 (1.6)	2 773 (2.1) 236 (0.2) 552 (0.4) 1 887 (1.4)	2 986 (3.7) 377 (0.5) 730 (0.9) 1 483 (1.8)
**Year of birth:**			
**1998–2001** **2002–2005** **2006–2009** **2010–2014**	47 107 (21.9) 48 596 (22.6) 53 052 (24.7) 66 079 (30.8)	28 817 (21.5) 30 789 (23.0) 33 269 (24.8) 41 209 (30.7)	18 290 (22.7) 17 807 (22.1) 19 783 (24.5) 24 870 (30.8)
**Season of birth:**			
**Winter** **spring** **Summer** **Fall**	49 039 (22.8) 56 517 (26.3) 58 987 (27.5) 50 291 (23.4)	30 678 (22.9) 34 935 (26.1) 36 831 (27.5) 31 640 (23.6)	18 361 (22.7) 21 582 (26.7) 22 156 (27.4) 18 651 (23.1)

^a^ Numbers (percentage). Percentages are calculated based on non-missing data

^b^ SEFI was categorized with cut off points within one standard deviation from the mean into high, middle, low middle and low SES

^c^ Defined as receiving income assistance for at least two months within 1 year before to 18 months after index date

^d^ Missing data for 886 (0.4%) subjects

^e^ Defined as the number of births following a multiple gestation pregnancy

^f^ Defined as having birth weight below the 10^th^ percentile for the gestational age and sex. Missing data for 502 (0.2%) subjects

^g^ Missing data for 109 491 (51.0%) subjects

^h^ Missing data for 107 550 (50.1%) subjects

^i^ Defined as having an infection code in physician claims only

^j^ Defined as having a hospitalization with an infection code

^k^ Defined as filling one or more antibiotic prescription during the first year of life

During a follow-up of 1 943 612 person-years with a median of 8.6 person-years (Interquartile range 4.8–13.2), 2965 children received an ASD diagnosis. The crude incidence rates for ASD diagnosis were 1.62 per 1000 person-years and 1.47 per 1000 person-years in children exposed and unexposed to antibiotics prenatally, respectively.

The sibling cohort included 75 896 subjects, with 53 840 exposure discordant pairs (Fig 1). In this cohort, 977 subjects developed ASD during a median follow up of 9.0 person-years (Interquartile range 5.5–12.9). Baseline characteristics of the sibling cohort are further described in S6 Table.

### Cox regression models

Prenatal antibiotic exposure was associated with a small increase in ASD risk (HR 1.11 [95% CI 1.03, 1.19]). After adjusting for covariates (Table 2), the risk estimates remained unchanged (HR 1.10 [95% CI 1.01, 1.19]). An interaction between antibiotics use and region was statistically significant (p-value = 0.02). The association with ASD risk was statistically significant in children residing in rural regions (HR 1.25 [95% CI 1.08, 1.44]), but not in those residing in urban regions (HR 1.02 [95% CI 0.92, 1.13]). In secondary analyses, statistically significant association was observed for those exposed to antibiotics in the second or third trimester (HR 1.11 [95% CI 1.01, 1.23] and 1.17 [95% CI 1.06, 1.30], respectively) or those exposed to penicillins or another beta-lactam (HR 1.13 [95% CI 1.04, 1.24] and 1.18 [95% CI 1.03, 1.37]), respectively). Analysis based on cumulative duration of antibiotic exposure showed a dose response effect, with the highest risk observed in those exposed to antibiotics for longer than 14 days (HR 1.15 [95% CI 1.01, 1.30]). In the sibling cohort, prenatal antibiotic exposure was associated with a small, non-statistically significant increase in the risk of ASD (HR 1.08 [95% CI 0.90, 1.30]). No substantial variation in the risk association was observed in all secondary analyses (Table 3).

**Table 2:** Association between antibiotic exposure and risk of ASD in the overall cohort.

Variable	Person-years	Number of events	HR [95% CI]	
			Unadjusted	Adjusted ^a^
**Main analysis**				
** Prenatal antibiotic exposure**	1 943 612	2 965	1.11 [1.03 , 1.19]**	1.10 [1.01 , 1.19]*
** Stratified by sex:**				
** Male**	991 939	2 401	1.11 [1.02 , 1.20]*	1.12 [1.02 , 1.22]*
** Female**	951 673	564	1.10 [0.93 , 1.30]	1.01 [0.84 , 1.23]
** Stratified by region:**				
** Rural**	887 497	953	1.28 [1.13 , 1.46]***	1.25 [1.08 , 1.44]**
** Urban**	1 056 105	2 012	1.06 [0.97 , 1.17]	1.02 [0.92 , 1.13]
**Exposure by trimester:** ^**b**^				
** None**	1 211 385	1 778	1.00 [Reference]	1.00 [Reference]
** First**	330 263	527	1.09 [0.99 , 1.20]	1.04 [0.94 , 1.16]
** Second**	372 136	620	1.14 [1.04 , 1.25]**	1.11 [1.01 , 1.23]*
** Third**	331 159	549	1.13 [1.03 , 1.2])*	1.17 [1.06 , 1.30]**
**Secondary analyses**				
** Antibiotic class**				
** None**	1 211 385	1 778	1.00 [Reference]	1.00 [Reference]
** Penicillin antibiotics**	494 406	813	1.12 [1.03 , 1.22]**	1.13 [1.04 , 1.24]**
** Other beta lactams**	132 014	233	1.19 [1.04 , 1.36]*	1.18 [1.03 , 1.37]*
** Macrolides and related antibiotics**	135 917	222	1.11 [0.97 , 1.28]	1.04 [0.90 , 1.20]
** Others**	194 826	302	1.06 [0.94 , 1.20]	1.01 [0.98 , 1.15]
** Number of antibiotic courses**				
** 0**	1 211 385	1 778	1.00 [Reference]	1.00 [Reference]
** 1**	458 378	739	1.10 [1.10 , 1.20]*	1.10 [1.00 , 1.20]
** 2**	166 695	266	1.09 [0.96 , 1.24]	1.07 [0.93 , 1.22]
** > = 3**	107 154	182	1.16 [1.00 , 1.35]	1.16 [0.98 , 1.37]
** Cumulative antibiotic duration (days)**				
** 0**	1 211 385	1 778	1.00 [Reference]	1.00 [Reference]
** 1–7**	300 554	472	1.07 [0.96 , 1.18]	1.07 [0.96 , 1.19]
** 8–14**	237 233	384	1.11 [0.99 , 1.24]	1.10 [0.98 , 1.24]
** >14**	194 441	331	1.17 [1.04 , 1.31]*	1.15 [1.01 , 1.30]*

P-value:

*P<0.05

**<P0.01

***P<0.001

^a^ Adjusted for sex, region, SES, maternal age at delivery, maternal medical conditions (mood and anxiety disorders, schizophrenia, DM, prenatal infections) prenatal antidepressants use, size for gestational age, childhood medical conditions (epilepsy, infections, neonatal jaundice, asthma and a diagnosis with other developmental disability disorder), antibiotics use in the first year of life, birth complications, mode of delivery, multiple birth, breastfeeding initiation, year of birth, season of birth, and birth order, health care access

^b^ First trimester = 0 to 13 weeks of pregnancy, second trimester = 14 to 27 weeks, third trimester = 28 weeks until date of birth

**Table 3:** Association between antibiotic exposure and risk of ASD in the sibling cohort.

Variable	Person-years	Number of events	HR [95% CI]	
			Unadjusted	Adjusted ^a^
**Main analysis**				
** Prenatal antibiotics exposure**	700 610	977	1.05 [0.90 ,1.22]	1.08 [0.90 , 1.30]
** Stratified by sex:**				
** Male**	357 076	786	1.21 [0.97 , 1.52]	1.14 [0.89 , 1.47]
** Female**	343 535	191	1.03 [0.64 , 1.64]	1.10 [0.57 , 2.11]
** Stratified by region:**				
** Rural**	361 486	378	1.12 [0.87 , 1.45]	1.19 [0.85 , 1.67]
** Urban**	339 124	599	1.07 [0.88 , 1.30]	1.07 [0.84 , 1.37]
**Exposure by trimester:** ^**b**^				
** None**	368 871	501	1.00 [Reference]	1.00 [Reference]
** First**	141 496	190	1.15 [0.94 , 1.41]	1.25 [1.00 , 1.58]
** Second**	159 418	249	1.13 [0.94 , 1.36]	1.16 [0.94 , 1.44]
** Third**	143 039	207	1.21 [0.99 , 1.47]	1.25 [1.00 , 1.57]
**Secondary analyses**				
** Antibiotic class**				
** None**	368 871	501	1.00 [Reference]	1.00 [Reference]
** Penicillin**	219 436	327	1.11 [0.94 , 1.32]	1.18 [0.97 , 1.45]
** Macrolides and related antibiotics**	59 611	93	1.27 [0.96 , 1.68]	1.24 [0.91 , 1.68]
** Other Beta lactams**	57 224	82	1.27 [0.95 , 1.71]	1.29 [0.94 , 1.77]
** Others**	80 109	101	0.96 [0.74 , 1.26]	1.01 [0.75 , 1.36]
** Number of antibiotic courses**				
** 0**	368 871	501	1.00 [Reference]	1.00 [Reference]
** 1**	226 323	311	0.99 [0.83 , 1.18]	1.04 [0.85 , 1.28]
** 2**	70 245	115	1.14 [0.86 , 1.51]	1.17 [0.84 , 1.62]
** > = 3**	35 171	50	1.33 [0.87 , 2.03]	1.20 [0.73 , 1.96]
** Cumulative antibiotic duration [days]**				
** 0**	368 871	501	1.00 [Reference]	1.00 [Reference]
** 1–7**	148 816	200	0.97 [0.78 , 1.19]	1.04 [0.81 , 1.32]
** 8–14**	111 409	165	1.03 [0.82 , 1.30]	1.06 [0.81 , 1.38]
** >14**	71 514	111	1.29 [0.96 , 1.72]	1.26 [0.89 , 1.79]

^a^ Adjusted for sex, region, SES, maternal age at delivery, prenatal infections, prenatal antidepressants use, size for gestational age, childhood medical conditions [epilepsy, infections, neonatal jaundice, asthma and a diagnosis with other developmental disability disorder], antibiotics use in the first year of life, birth complications, mode of delivery, multiple birth, breastfeeding initiation, year of birth, season of birth, and birth order.

^b^ First trimester = 0 to 13 weeks of pregnancy, second = 13 to 27 weeks, third = 28 weeks until date of birth

### Sensitivity analyses

**Fig 2:**
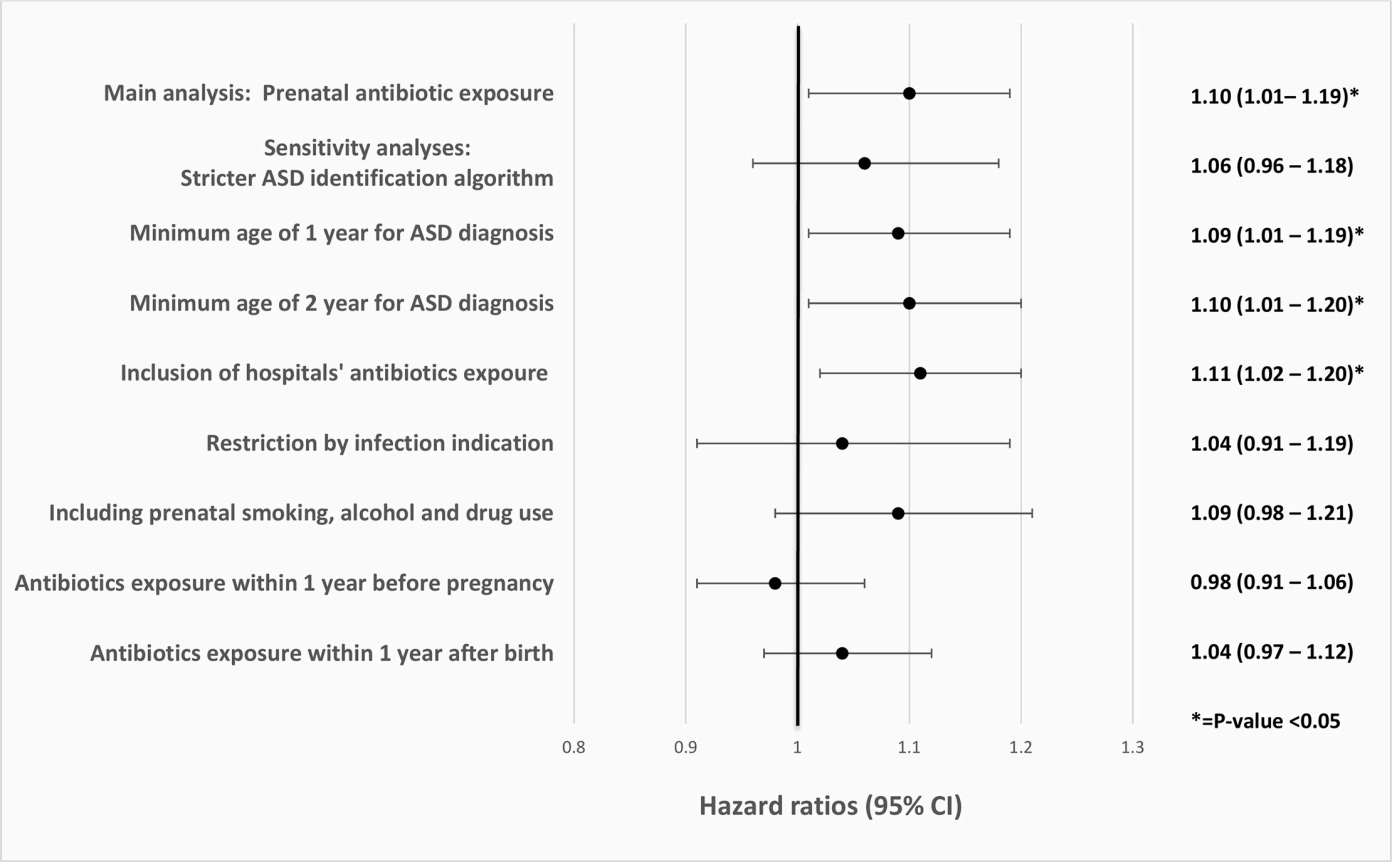
Forest plot with sensitivity analyses risk estimates.

The association between prenatal antibiotic exposure and ASD was consistent across the different sensitivity analyses (Fig 2). The risk ranged from HR 1.04 [95% CI 0.91, 1.19] when restricting the cohort to those who had an infection diagnostic code during pregnancy to HR 1.11 [95% CI 1.02, 1.20] when including inpatient antibiotic use. Maternal antibiotic exposure in the year before pregnancy and the year after birth were not found to be associated with ASD risk in the child (HR 0.98 [95% CI 0.91, 1.06] and 1.03 [95% CI 0.94, 1.12], respectively).

## Discussion

Findings from this large population-based cohort study showed a 10% increase in the risk of ASD in children exposed to antibiotics prenatally compared to those who were not exposed. This association was dependent on region and was only observed in those residing in rural regions. The increased risk was shown in those exposed to penicillins and other beta lactams and in those exposed to antibiotics in the second or third trimester. The highest risk was observed in those exposed to antibiotics for longer than two weeks or who received 3 or more antibiotic courses. The lack of association in the two negative controls provides confidence that the findings are reliable.

Since the main model could not account for all confounding sources, we explored the association using a sibling cohort design to address environmental, genetic and other familial or social factors. In the analysis of the sibling cohort, the risk of ASD with prenatal antibiotic exposure did not change significantly, except the association was no longer statistically significant. This could be explained by the smaller sample size of the sibling cohort. This led us to conclude that prenatal antibiotic exposure appears to be associated with a small increase in ASD risk. Nevertheless, we believe the observed risk is too small to be clinically meaningful and may have been influenced by residual confounding from variables that could not be identified in the Repository, not shared by sibling pairs or not recorded correctly.

Study findings are consistent with a previous exploratory population-based cohort study conducted by Atladóttir HO et al in Denmark [29]. The study investigated the association of self-reported maternal infections, febrile episodes and prenatal antibiotics use with the risk for ASD. There was no association of maternal infection or febrile episodes with the risk for ASD. However, there was an increased risk of ASD with prenatal use of antibiotics (HR 1.20 [95% CI 1.00, 1.40]). Due to the exploratory nature of this study, and the potential misclassification of the self-reported exposure data, the findings needed to be replicated in a large study designed to address this research question using reliable exposure data sources. Even though the study by Atladóttir HO et al. did not find an association between prenatal infection and ASD risk, other studies reported increased ASD risk with multiple prenatal infections or infections requiring hospitalization [30, 31]. Accordingly, confounding by indication is a concern and may have influenced the findings of the current study.

Our study has several strengths including the large sample size, long follow-up period, the use of administrative databases to identify the exposure and the outcome, including many potential confounders and the sibling-controlled design that minimized confounding by unmeasured familial factors. Despite the mentioned strengths, few limitations need to be considered. Exposure misclassification is a concern since drug dispensation does not necessarily indicate drug use. In addition, inpatient antibiotic dispensations were not included in the main analysis due to the limited data years and geographic coverage of the inpatient dispensation database. However, the observed association did not change significantly in the sensitivity analysis that included the subset of data available on inpatient dispensations. Outcome misclassification is another concern given that the utilized ASD identification algorithm has not been independently validated, yet using a stricter ASD identification algorithm did not change the risk estimate. The potential for unmeasured confounding from variables that are not shared by sibling pairs is a potential limitation. For example, we could only identify maternal siblings due to the lack of reliable linked data identifying the fathers. Hence, the sibling cohort included both full and half siblings, which is not ideal to account for confounding by genetic factors. Confounding by indication is another limitation and may have influenced study findings. Future studies are needed to explore other methods to control for confounding in examining similar associations. In addition, studies are recommended to investigate antibiotic-induced microbial dysbiosis and microbiota involvement in neurodevelopmental disorders at the biological level to shed light on the etiology of these disorders and inform disease prevention.

## Conclusion

Our results suggest that prenatal antibiotic exposure is associated with a small, albeit clinically non-significant increase in the risk of ASD which may have been influenced by unmeasured confounding.

## Supporting information

S1 Table Description and years of data sources. (DOCX)

S2 Table Antibiotics classification according to Anatomical Therapeutic Chemical (ATC). (DOCX)

S3 Table Prenatal medications explored as covariates. (DOCX)

S4 Table Identification algorithm of medical conditions. (DOCX)

S5 Table Description of antibiotics use. (DOCX)

S6 Table Characteristics of sibling cohort: Overall and by antibiotics exposure status. (DOCX)
