# Supplementary material for: Prenatal antibiotics exposure and the risk of autism spectrum disorders: A population-based cohort study
Source: PLoS One. 2019 Aug 29;14(8):e0221921. doi: 10.1371/journal.pone.0221921 (PMC6715235; doi:10.1371/journal.pone.0221921)
Supplement: S1 Table — (DOCX) [file pone.0221921.s001.docx]

**S1 Table. Description and years of data sources**

| **Database** | **Description** | **Data years** |
| --- | --- | --- |
| **Drug Program Information Network (DPIN)** | An electronic drug system that captures all prescriptions dispensed from community pharmacies to residents and is maintained by Manitoba Health, Seniors and Active Living. It includes prescription information such as generic name, Anatomical Therapeutic Chemical (ATC) code, date of dispensation, strength and days supplied. | 1995/96 - 2015/16 |
| **In-hospital Pharmaceuticals** | An inpatient pharmacy system that provides dispensing information on pharmaceutical use at three Winnipeg hospitals: Health Sciences Centre, Concordia Hospital and Riverview Health Centre. | 1999-2012 |
| **Hospital discharge abstracts** | Includes records of all patients’ hospital admissions with summaries for demographic data such as gender and postal code, and clinical data including up to 25 diagnosis codes and 20 procedure codes. | 1970/71 - 2015/16 |
| **Medical Services database** | Physician claims in the Medical Services database include records of claims for any physician visits in offices, hospitals and outpatient departments in Manitoba in addition to some information on Manitobans’ physician visits outside the province. Claims are submitted by physicians electronically for service reimbursement by Manitoba Health, Seniors and Active Living. | 1970/71 - 2015/16 |
| **The Manitoba Education and Training Special Needs funding data file** | Includes education records on special school funding received for students with special needs, including those with ASD. | 1995/96 - 2015/16 |
| **The Hospital Newborn to Mother Link** | Serves to match the baby’s birth hospital record with the mother's obstetrical delivery record and contains basic demographic and hospital data on newborns and their mothers for in-hospital births. | 1984/85 - 2015/16 |
| **BabyFirst - Families First Screen** | Collected by Public Health Nurses on nearly all families with newborns in Manitoba, and maintained by Heathy Child Manitoba. Contains records of newborns identifying biological, social, and demographic risk factors including prenatal smoking, alcohol and drug use. | 2000 – 2013 |
| **The Social Allowances Management Information Network** | Provides information concerning employment and income assistance received by Manitoba residents. | 1995/96 - 2014/15 |
| **Canada Census** | A population survey with aggregate demographic information for all persons within a dissemination area in Canada and is conducted by Statistics Canada every five years. | 1971 – 2016 |
