# Supplementary material for: Prenatal antibiotics exposure and the risk of autism spectrum disorders: A population-based cohort study
Source: PLoS One. 2019 Aug 29;14(8):e0221921. doi: 10.1371/journal.pone.0221921 (PMC6715235; doi:10.1371/journal.pone.0221921)
Supplement: S2 Table — (DOCX) [file pone.0221921.s002.docx]

**S2 Table. Antibiotics classification according to Anatomical Therapeutic Chemical (ATC)**

| **Class** | **Generic name** | **ATC** |
| --- | --- | --- |
| **Penicillin Beta-lactams** | Amoxicillin, ampicillin, bacampicillin, penicillin, piperacillin, pivampicillin, pivmecillinam, cloxacillin, floxacillin, nafcillin, ticarcillin, tazobactam | J01C |
| **Other Beta-lactams** | Cefazolin, cefepime, cefixime, cefotaxime, cefotetan, cefoxitin, cefpodoxime, cefprozil, ceftazidime, ceftizoxime, ceftobiprole, ceftriaxone, cefuroxime, cephalexin, cephalothin, aztreonam, cefaclor, cefadroxil, cefamandole, doripenem, ertapenem, meropenem | J01D |
| **Macrolides and related antibiotics** | Dalfopristin, telithromycin, azithromycin, clarithromycin, clindamycin, erythromycin, lincomycin | J01F, J01R |
| **Other antibiotics** | Minocycline, doxycycline, demeclocycline, rolitetracycline, tetracycline, ciprofloxacin, gatifloxacin, nalidixic acid, gemifloxacin, grepafloxacin, levofloxacin, moxifloxacin, norfloxacin, ofloxacin, trovafloxacin, methenamine, fosfomycin, tedizolid, nitrofurantoin, metronidazole, linezolid, fusidate, fusidic acid, bacitracin, vancomycin, colistin, polymyxin B, telavancin, tobramycin, gentamicin, daptomycin, amikacin, spectinomycin, neomycin, netilmicin, spiramycin, streptomycin, chloramphenicol, trimethoprim, sulfamethoxazole, sulfadiazine, sulfapyridine, sulfisoxazole | J01A, J01B, J01G, J01E, J01M, J01X |
