# Supplementary material for: Prenatal antibiotics exposure and the risk of autism spectrum disorders: A population-based cohort study
Source: PLoS One. 2019 Aug 29;14(8):e0221921. doi: 10.1371/journal.pone.0221921 (PMC6715235; doi:10.1371/journal.pone.0221921)
Supplement: S3 Table — (DOCX) [file pone.0221921.s003.docx]

**S3 Table. Prenatal medications explored as covariates ^a^**

| **Class** | **Generic name** | **ATC** |
| --- | --- | --- |
| **Antidepressants** | [Desipramine](https://www.whocc.no/atc_ddd_index/?code=N06AA01&showdescription=yes), [imipramine](https://www.whocc.no/atc_ddd_index/?code=N06AA02&showdescription=yes), [clomipramine](https://www.whocc.no/atc_ddd_index/?code=N06AA04&showdescription=yes) , [trimipramine](https://www.whocc.no/atc_ddd_index/?code=N06AA06&showdescription=yes), [amitriptyline](https://www.whocc.no/atc_ddd_index/?code=N06AA09&showdescription=yes), [nortriptyline](https://www.whocc.no/atc_ddd_index/?code=N06AA10&showdescription=yes), [protriptyline](https://www.whocc.no/atc_ddd_index/?code=N06AA11&showdescription=yes), [doxepin](https://www.whocc.no/atc_ddd_index/?code=N06AA12&showdescription=yes), [amoxapine](https://www.whocc.no/atc_ddd_index/?code=N06AA17&showdescription=yes), [amineptine](https://www.whocc.no/atc_ddd_index/?code=N06AA19&showdescription=yes), [maprotiline](https://www.whocc.no/atc_ddd_index/?code=N06AA21&showdescription=yes), [fluoxetine](https://www.whocc.no/atc_ddd_index/?code=N06AB03&showdescription=yes), [citalopram](https://www.whocc.no/atc_ddd_index/?code=N06AB04&showdescription=yes), [paroxetine](https://www.whocc.no/atc_ddd_index/?code=N06AB05&showdescription=yes), [sertraline](https://www.whocc.no/atc_ddd_index/?code=N06AB06&showdescription=yes), [fluvoxamine](https://www.whocc.no/atc_ddd_index/?code=N06AB08&showdescription=yes), [escitalopram](https://www.whocc.no/atc_ddd_index/?code=N06AB10&showdescription=yes), [phenelzine](https://www.whocc.no/atc_ddd_index/?code=N06AF03&showdescription=yes), [tranylcypromine](https://www.whocc.no/atc_ddd_index/?code=N06AF04&showdescription=yes), [moclobemide](https://www.whocc.no/atc_ddd_index/?code=N06AG02&showdescription=yes), [tryptophan](https://www.whocc.no/atc_ddd_index/?code=N06AX02&showdescription=yes), [trazodone](https://www.whocc.no/atc_ddd_index/?code=N06AX05&showdescription=yes), [nefazodone](https://www.whocc.no/atc_ddd_index/?code=N06AX06&showdescription=yes), [mirtazapine](https://www.whocc.no/atc_ddd_index/?code=N06AX11&showdescription=yes), [bupropion](https://www.whocc.no/atc_ddd_index/?code=N06AX12&showdescription=yes), [venlafaxine](https://www.whocc.no/atc_ddd_index/?code=N06AX16&showdescription=yes), [milnaacipran](https://www.whocc.no/atc_ddd_index/?code=N06AX17&showdescription=yes), [duloxetine](https://www.whocc.no/atc_ddd_index/?code=N06AX21&showdescription=yes), [desvenlafaxine](https://www.whocc.no/atc_ddd_index/?code=N06AX23&showdescription=yes), [vilazodone](https://www.whocc.no/atc_ddd_index/?code=N06AX24&showdescription=yes), [vortioxetine](https://www.whocc.no/atc_ddd_index/?code=N06AX26&showdescription=yes). | N06A |
| **Cardiovascular medications** | Antihypertensive agents: [reserpine](https://www.whocc.no/atc_ddd_index/?code=C02AA02&showdescription=yes), methyldopa, [clonidine](https://www.whocc.no/atc_ddd_index/?code=C02AC01&showdescription=yes), [guanfacine](https://www.whocc.no/atc_ddd_index/?code=C02AC02&showdescription=yes), [prazosin](https://www.whocc.no/atc_ddd_index/?code=C02CA01&showdescription=yes), [doxazosin](https://www.whocc.no/atc_ddd_index/?code=C02CA04&showdescription=yes), [guanethidine](https://www.whocc.no/atc_ddd_index/?code=C02CC02&showdescription=yes), [diazoxide](https://www.whocc.no/atc_ddd_index/?code=C02DA01&showdescription=yes), [hydralazine](https://www.whocc.no/atc_ddd_index/?code=C02DB02&showdescription=yes), [minoxidil](https://www.whocc.no/atc_ddd_index/?code=C02DC01&showdescription=yes), [nitroprusside](https://www.whocc.no/atc_ddd_index/?code=C02DD01&showdescription=yes), [bosentan](https://www.whocc.no/atc_ddd_index/?code=C02KX01&showdescription=yes), [ambrisentan](https://www.whocc.no/atc_ddd_index/?code=C02KX02&showdescription=yes), [macitentan](https://www.whocc.no/atc_ddd_index/?code=C02KX04&showdescription=yes), [riociguat](https://www.whocc.no/atc_ddd_index/?code=C02KX05&showdescription=yes).  Peripheral vasodilators: [phentolamine](https://www.whocc.no/atc_ddd_index/?code=C04AB01&showdescription=yes), [nicotinic acid](https://www.whocc.no/atc_ddd_index/?code=C04AC01&showdescription=yes), [pentoxifylline](https://www.whocc.no/atc_ddd_index/?code=C04AD03&showdescription=yes), ergoloid mesylates, [cyclandelate](https://www.whocc.no/atc_ddd_index/?code=C04AX01&showdescription=yes).  Beta blockers: [oxprenolol](https://www.whocc.no/atc_ddd_index/?code=C07AA02&showdescription=yes), [pindolol](https://www.whocc.no/atc_ddd_index/?code=C07AA03&showdescription=yes), [propranolol](https://www.whocc.no/atc_ddd_index/?code=C07AA05&showdescription=yes), [timolol](https://www.whocc.no/atc_ddd_index/?code=C07AA06&showdescription=yes), [sotalol](https://www.whocc.no/atc_ddd_index/?code=C07AA07&showdescription=yes), [nadolol](https://www.whocc.no/atc_ddd_index/?code=C07AA12&showdescription=yes), [metoprolol](https://www.whocc.no/atc_ddd_index/?code=C07AB02&showdescription=yes), [atenolol](https://www.whocc.no/atc_ddd_index/?code=C07AB03&showdescription=yes), [acebutolol](https://www.whocc.no/atc_ddd_index/?code=C07AB04&showdescription=yes), [bisoprolol](https://www.whocc.no/atc_ddd_index/?code=C07AB07&showdescription=yes), [esmolol](https://www.whocc.no/atc_ddd_index/?code=C07AB09&showdescription=yes), [nebivolol](https://www.whocc.no/atc_ddd_index/?code=C07AB12&showdescription=yes), [labetalol](https://www.whocc.no/atc_ddd_index/?code=C07AG01&showdescription=yes), [carvedilol](https://www.whocc.no/atc_ddd_index/?code=C07AG02&showdescription=yes).  Calcium channel blockers: [amlodipine](https://www.whocc.no/atc_ddd_index/?code=C08CA01&showdescription=yes), [felodipine](https://www.whocc.no/atc_ddd_index/?code=C08CA02&showdescription=yes), [nicardipine](https://www.whocc.no/atc_ddd_index/?code=C08CA04&showdescription=yes), [nifedipine](https://www.whocc.no/atc_ddd_index/?code=C08CA05&showdescription=yes), [nimodipine](https://www.whocc.no/atc_ddd_index/?code=C08CA06&showdescription=yes), [clevidipine](https://www.whocc.no/atc_ddd_index/?code=C08CA16&showdescription=yes), [verapamil](https://www.whocc.no/atc_ddd_index/?code=C08DA01&showdescription=yes), [diltiazem](https://www.whocc.no/atc_ddd_index/?code=C08DB01&showdescription=yes).  Agents acting on the renin-angiotensin system: [captopril](https://www.whocc.no/atc_ddd_index/?code=C09AA01&showdescription=yes), [enalapril](https://www.whocc.no/atc_ddd_index/?code=C09AA02&showdescription=yes), [lisinopril](https://www.whocc.no/atc_ddd_index/?code=C09AA03&showdescription=yes), [perindopril](https://www.whocc.no/atc_ddd_index/?code=C09AA04&showdescription=yes), [ramipril](https://www.whocc.no/atc_ddd_index/?code=C09AA05&showdescription=yes), [quinapril](https://www.whocc.no/atc_ddd_index/?code=C09AA06&showdescription=yes), [benazepril](https://www.whocc.no/atc_ddd_index/?code=C09AA07&showdescription=yes), [cilazapril](https://www.whocc.no/atc_ddd_index/?code=C09AA08&showdescription=yes), [fosinopril](https://www.whocc.no/atc_ddd_index/?code=C09AA09&showdescription=yes), [trandolapril](https://www.whocc.no/atc_ddd_index/?code=C09AA10&showdescription=yes), [losartan](https://www.whocc.no/atc_ddd_index/?code=C09CA01&showdescription=yes), [eprosartan](https://www.whocc.no/atc_ddd_index/?code=C09CA02&showdescription=yes), [valsartan](https://www.whocc.no/atc_ddd_index/?code=C09CA03&showdescription=yes), [irbesartan](https://www.whocc.no/atc_ddd_index/?code=C09CA04&showdescription=yes), [candesartan](https://www.whocc.no/atc_ddd_index/?code=C09CA06&showdescription=yes), [telmisartan](https://www.whocc.no/atc_ddd_index/?code=C09CA07&showdescription=yes), [olmesartan, azilsartan, [aliskiren](https://www.whocc.no/atc_ddd_index/?code=C09XA02&showdescription=yes).](https://www.whocc.no/atc_ddd_index/?code=C09CA08&showdescription=yes) | C02  C04  C07  C08  C09 |
| **Antipsychotics** | [Chlorpromazine](https://www.whocc.no/atc_ddd_index/?code=N05AA01&showdescription=yes), [promazine](https://www.whocc.no/atc_ddd_index/?code=N05AA03&showdescription=yes), [fluphenazine](https://www.whocc.no/atc_ddd_index/?code=N05AB02&showdescription=yes), [perphenazine](https://www.whocc.no/atc_ddd_index/?code=N05AB03&showdescription=yes), [prochlorperazine](https://www.whocc.no/atc_ddd_index/?code=N05AB04&showdescription=yes), [trifluoperazine](https://www.whocc.no/atc_ddd_index/?code=N05AB06&showdescription=yes), [thioproperazine](https://www.whocc.no/atc_ddd_index/?code=N05AB08&showdescription=yes), [perazine](https://www.whocc.no/atc_ddd_index/?code=N05AB10&showdescription=yes), [periciazine](https://www.whocc.no/atc_ddd_index/?code=N05AC01&showdescription=yes), [thioridazine](https://www.whocc.no/atc_ddd_index/?code=N05AC02&showdescription=yes), [mesoridazine](https://www.whocc.no/atc_ddd_index/?code=N05AC03&showdescription=yes), [pipotiazine](https://www.whocc.no/atc_ddd_index/?code=N05AC04&showdescription=yes), [haloperidol](https://www.whocc.no/atc_ddd_index/?code=N05AD01&showdescription=yes), [droperidol](https://www.whocc.no/atc_ddd_index/?code=N05AD08&showdescription=yes), [ziprasidone](https://www.whocc.no/atc_ddd_index/?code=N05AE04&showdescription=yes), [lurasidone](https://www.whocc.no/atc_ddd_index/?code=N05AE05&showdescription=yes), flupentixol, [clopenthixol](https://www.whocc.no/atc_ddd_index/?code=N05AF02&showdescription=yes), [zuclopenthixol](https://www.whocc.no/atc_ddd_index/?code=N05AF05&showdescription=yes), [fluspirilene](https://www.whocc.no/atc_ddd_index/?code=N05AG01&showdescription=yes), [pimozide](https://www.whocc.no/atc_ddd_index/?code=N05AG02&showdescription=yes), [loxapine](https://www.whocc.no/atc_ddd_index/?code=N05AH01&showdescription=yes), [clozapine](https://www.whocc.no/atc_ddd_index/?code=N05AH02&showdescription=yes), [olanzapine](https://www.whocc.no/atc_ddd_index/?code=N05AH03&showdescription=yes), [quetiapine](https://www.whocc.no/atc_ddd_index/?code=N05AH04&showdescription=yes), [asenapine](https://www.whocc.no/atc_ddd_index/?code=N05AH05&showdescription=yes), [lithium](https://www.whocc.no/atc_ddd_index/?code=N05AN01&showdescription=yes), [risperidone](https://www.whocc.no/atc_ddd_index/?code=N05AX08&showdescription=yes), [aripiprazole](https://www.whocc.no/atc_ddd_index/?code=N05AX12&showdescription=yes), [paliperidone](https://www.whocc.no/atc_ddd_index/?code=N05AX13&showdescription=yes), [brexpiprazole](https://www.whocc.no/atc_ddd_index/?code=N05AX16&showdescription=yes). | N05A |
| **Anticonvulsants** | [Phenobarbital](https://www.whocc.no/atc_ddd_index/?code=N03AA02&showdescription=yes), [primidone](https://www.whocc.no/atc_ddd_index/?code=N03AA03&showdescription=yes), [phenytoin](https://www.whocc.no/atc_ddd_index/?code=N03AB02&showdescription=yes), [fosphenytoin](https://www.whocc.no/atc_ddd_index/?code=N03AB05&showdescription=yes), [ethosuximide](https://www.whocc.no/atc_ddd_index/?code=N03AD01&showdescription=yes), [clonazepam](https://www.whocc.no/atc_ddd_index/?code=N03AE01&showdescription=yes), carbamazepine, [oxcarbazepine](https://www.whocc.no/atc_ddd_index/?code=N03AF02&showdescription=yes), [rufinamide](https://www.whocc.no/atc_ddd_index/?code=N03AF03&showdescription=yes), [eslicarbazepine](https://www.whocc.no/atc_ddd_index/?code=N03AF04&showdescription=yes), [valproic acid](https://www.whocc.no/atc_ddd_index/?code=N03AG01&showdescription=yes), [vigabatrin](https://www.whocc.no/atc_ddd_index/?code=N03AG04&showdescription=yes), [lamotrigine](https://www.whocc.no/atc_ddd_index/?code=N03AX09&showdescription=yes), [topiramate](https://www.whocc.no/atc_ddd_index/?code=N03AX11&showdescription=yes), [gabapentin](https://www.whocc.no/atc_ddd_index/?code=N03AX12&showdescription=yes), [levetiracetam](https://www.whocc.no/atc_ddd_index/?code=N03AX14&showdescription=yes), [pregabalin](https://www.whocc.no/atc_ddd_index/?code=N03AX16&showdescription=yes), [stiripentol](https://www.whocc.no/atc_ddd_index/?code=N03AX17&showdescription=yes), [lacosamide](https://www.whocc.no/atc_ddd_index/?code=N03AX18&showdescription=yes), [perampanel](https://www.whocc.no/atc_ddd_index/?code=N03AX22&showdescription=yes), [brivaracetam](https://www.whocc.no/atc_ddd_index/?code=N03AX23&showdescription=yes), [beclamide](https://www.whocc.no/atc_ddd_index/?code=N03AX30&showdescription=yes). | N03A |
| ^a^: Two prescription fills during pregnancy was required. | | |
