# Supplementary material for: Prenatal antibiotics exposure and the risk of autism spectrum disorders: A population-based cohort study
Source: PLoS One. 2019 Aug 29;14(8):e0221921. doi: 10.1371/journal.pone.0221921 (PMC6715235; doi:10.1371/journal.pone.0221921)
Supplement: S4 Table — (DOCX) [file pone.0221921.s004.docx]

**S4 Table. Identification algorithm of medical conditions**

| **Covariate** | **Period** | **Diagnostic Criteria** |
| --- | --- | --- |
| **Maternal medical conditions:** | | |
| **Mood and anxiety disorders** | Within 3 years before index date | - One or more hospitalizations with a diagnosis for depressive disorder, affective psychoses, neurotic depression or adjustment reaction: ICD-9-CM codes 296.1-296.8, 300.4, 309 or 311; ICD-10-CA codes F31, F32, F33, F34.1, F38.0, F38.1, F41.2, F43.1, F43.2, F43.8, F53.0, F93.0 or with a diagnosis for an anxiety state, phobic disorders or obsessive-compulsive disorders: ICD-9-CM codes 300.0, 300.2, 300.3, 300.7; ICD-10-CA codes F40, F41.0, F41.1, F41.3, F41.8, F41.9, F42, F45.2, OR - One or more hospitalizations with a diagnosis for anxiety disorders: ICD-9-CM code 300; ICD-10-CA codes F32, F34.1, F40, F41, F42, F44, F45.0, F45.1, F45.2, F48, F68.0, or F99 AND one or more prescriptions for an antidepressant or mood stabilizer, including medications with the ATC codes N05AN01, N05BA, N06A, OR - One or more physician visits with a diagnosis for depressive disorder or affective psychoses: ICD-9-CM codes 296, 311, OR - One or more physician visits with a diagnosis for anxiety disorders: ICD-9-CM code 300 AND one or more prescriptions for an antidepressant or mood stabilizer, including medications with the ATC codes N05AN01, N05BA, N06A, OR - Three or more physician visits with a diagnosis for anxiety disorders or adjustment reaction: ICD-9-CM code 300, 309. |
| **Schizophrenia** | Within 3 years before index date | - One or more hospitalization with a diagnosis for schizophrenia: ICD-9-CM code 295; ICD-10-CA codes F20, F21, F23.2, F25, OR - One or more physician visits with a diagnosis for schizophrenia: ICD-9-CM code 295. |
| **Diabetes mellitus** | Within 1 year before index date | - - One or more hospitalizations with a diabetes diagnosis: ICD-9-CM code 250, 648.0, 648.8; ICD-10-CA codes E10-E14, O24, OR   - Two or more physician visits with a diabetes diagnosis: ICD-9-CM code 250, OR   - One or more prescriptions for a diabetes medication (ATC: A10A, A10B). |
| **Prenatal infection** | During pregnancy | A hospitalization or physician visit with any of the following codes:  Eye and ear infections: ICD-9-CM codes 370*, 372*, 380*, 381*, 382; ICD-10-CA codes H10, H13.1, H16, H19.1, H19.2, H60, H62, H65, H66, H67. Upper respiratory tract infection: ICD-9-CM codes 460–465; ICD-10-CA codes J00-J06. Lower respiratory tract infection: ICD-9-CM codes 466, 481–488; ICD-10-CA codes J09- J18, J20-J22. Genitourinary system infection: ICD-9-CM codes 590, 595, 597, 599*, 601, 604, 614-617, 771*; ICD-10-CA codes N10-N12, N30, N33.0, N33.8, N37.0, N39.0, N41, N45, N70-N77.Central nervous system infections: ICD-9-CM codes 320, 321, 323, 324, 728*; ICD-10-CA codes G00-G02, G04-G07  Skin infections: ICD-9-CM codes 680-686; ICD-10-CA codes L00- L08, M72.6. Cardiovascular system infections: ICD-9-CM codes 391, 420, 421, 422, 424*; ICD-10-CA codes I01, I30, I32.0, I32.1, I33, I38, I39.8, I40, I41.0, I41.1, I41.2. |
| **Prenatal infection** | During pregnancy | Musculoskeletal system infections: ICD-9-CM codes 711, 730; ICD-10-CA codes M00- M01, M03, M46.2, M86  Bacteremia/Septicemia: ICD-9-CM codes 771*, 790*, 995*; ICD-10-CA codes P36, A22.7, A26.7, A02.1, A32.7, A02.1, A32.7, A40, A41, A42.7, B37.7  Gastrointestinal system infections: ICD-9-CM codes 535, 540, 541, 542, 566, 567, 530*, 572*, 575*; ICD-10-CA codes K20, K29.0, K29.1, K29.8, K35, K61, K65, K67, K81.0  Other Parasitic and infectious diseases: ICD-9-CM codes 001-139; ICD-10-CA codes A00-B99 |
| **Childhood medical conditions:** | | |
| **Birth complications** | At birth | Include vacuum, forceps or breech procedures: ICD-9-CM procedure codes 72, 73.3, 763.2, 652.2, 669.6; CCI codes 5MD53, 5MD54, 5MD55, 5MD56, shoulder dystocia: 660.4, placental abruption: 641.2, cord prolapse: 663, uterine rupture: 665.1 or other perinatal complications: 760-779. |
| **Epilepsy** | Within the first year of life | - One or more hospitalization for seizure disorder: ICD-9-CM codes 345, 649.4; ICD-10-CA codes G40, G41, OR - One or more physician visit for seizure disorder: ICD-9-CM code 345, OR - One or more prescription for an anticonvulsant medication (ATC: N03A) |
| **Asthma** | Within the first year of life | - - - One or more hospitalizations with a diagnosis of asthma: ICD-9-CM codes 493; ICD-10-CA code J45, OR     - One or more physician visits with a diagnosis of asthma: ICD-9-CM codes 493, OR     - One or more prescriptions for asthma medication (ATC: R03, R06AX17) |
| **Other developmental disabilities** | Within the first year of life | - One or more hospitalizations with diagnoses for intellectual disabilities, Down's syndrome, autosomal deletion syndromes, Prader-Willi syndrome, other specified congenital anomalies, or fetal alcohol syndrome: ICD-9-CM codes 317, 318, 319, 758.0- 758.3, 759.8, 760.71; ICD-10-CA: F70, F71, F72, F73, F78, F79, P04.3, Q86, Q87, Q89.8, Q90, Q91, Q93, Q99.2. - One or more physician visit with diagnoses for intellectual disabilities: ICD-9-CM: 317, 318, 319 |
| **Neonatal jaundice** | Within the first 4 weeks of life | - One or more hospitalizations with diagnoses for Jaundice: ICD-9-CM code 774; ICD–10–CA codes P58, P59, OR - One or more physician visit with diagnoses for Jaundice: ICD-9-CM code 774 |
| **Infection** | Within the first year of life | Similar to maternal definition |
