# Supplementary material for: Prenatal antibiotics exposure and the risk of autism spectrum disorders: A population-based cohort study
Source: PLoS One. 2019 Aug 29;14(8):e0221921. doi: 10.1371/journal.pone.0221921 (PMC6715235; doi:10.1371/journal.pone.0221921)
Supplement: S5 Table — (DOCX) [file pone.0221921.s005.docx]

**S5 Table. Description of antibiotic use**

|  | **Number (%)** |
| --- | --- |
| **Number of courses** | |
| 1 | 50 685 (62.8) |
| 2 | 18 427 (22.8) |
| >=3 | 11 638 (14.4) |
| Total | 80 750 |
| **Cumulative duration (days)** | |
| 1-7 | 34 213 (42.4) |
| 8-14 | 25 593 (31.7) |
| >14 | 20 944 (25.9) |
| Total | 80 750 |
| **Antibiotic class** | |
| Penicillin antibiotics | 53 232 (50.2) |
| Other beta lactams | 16 321 (15.4) |
| Macrolides and related antibiotics | 14 799 (14.0) |
| Others | 21 687 (20.5) |
| Total | 106 039 |
| **Trimester** |  |
| 1 | 36 563 (32.1) |
| 2 | 41 368 (36.3) |
| 3 | 35 907 (31.5) |
| Total | 113 838 |
