# Supplementary material for: Prenatal antibiotics exposure and the risk of autism spectrum disorders: A population-based cohort study
Source: PLoS One. 2019 Aug 29;14(8):e0221921. doi: 10.1371/journal.pone.0221921 (PMC6715235; doi:10.1371/journal.pone.0221921)
Supplement: S6 Table — (DOCX) [file pone.0221921.s006.docx]

**S6 Table. Characteristics of sibling cohort: overall and by antibiotics exposure status ^a^**

|  | **All subjects**  **N=75 896** | **Antibiotic use during pregnancy** | |
| --- | --- | --- | --- |
|  |  | **No**  **(N= 39 577)** | **Yes**  **(N= 36 319)** |
| **Male** | 38 788 (51.1) | 20 186 (51.0) | 18 602 (51.2) |
| **Urban region** | 36 648 (48.3) | 18 688 (47.2) | 17 960 (49.5) |
| **Socioeconomic status (SES)^b^:**  **High**  **Middle**  **Low-mid**  **Low** | 5 913 (7.8)  24 463 (32.2)  23 604 (31.1)  21 916 (28.9) | 3 051 (7.7)  12 849 (32.5)  12 160 (30.7)  11 517 (29.1) | 2 862 (7.9)  11 614 (32.0)  11 444 (31.5)  10 399 (28.6) |
| **Receipt of income assistance ^c^** | 16 779 (22.1) | 7 953 (20.1) | 8 826 (24.3) |
| **Mothers age at delivery:**  **< 30**  **30-39**  **>= 40** | 50 132(66.1)  24 780 (32.7)  984 (1.3) | 26 242 (66.3)  12 839 (32.4)  496 (1.3) | 23 890 (65.8)  11 941 (32.9)  488 (1.3) |
| **Breastfeeding initiation ^d^** | 58 576 (77.5) | 30 635 (77.8) | 27 941 (77.2) |
| **Multiple birth ^e^** | 1 548 (2.0) | 747 (1.9) | 801 (2.2) |
| **Caesarian section** | 13 178 (17.4) | 6 701 (16.9) | 6 477 (17.8) |
| **Birth complications** | 5 582 (7.4) | 2 905 (7.3) | 2 677 (7.4) |
| **First born child** | 21 037 (27.7) | 11 419 (28.9) | 9 618 (26.5) |
| **Small for gestational age ^f^** | 5 141 (6.8) | 2 682 (6.8) | 2 459 (6.8) |
| **Prenatal alcohol/drug use ^g^** | 5 279 (13.5) | 2 679 (13.1) | 2 600 (13.9) |
| **Prenatal smoking ^h^** | 8 881 (22.3) | 4 322 (20.8) | 4 559 (23.9) |
| **Childhood medical conditions:**  **Infections:**  **None**  **Mild-moderate ^i^**  **Severe ^j^**  **Epilepsy**  **Neonatal jaundice**  **Other developmental disabilities**  **Asthma** | 21 731 (28.6)  48 736 (64.2)  5 429 (7.2)  387 (0.5)  7 004 (9.2)  330 (0.4)  10 817 (14.3) | 12 184 (30.8)  24 541 (62.0)  2 852 (7.2)  204 (0.5)  3 615 (9.1)  159 (0.4)  5 232 (13.2) | 9 547 (26.3)  24 195 (66.6)  2 577 (7.1)  183 (0.5)  3 389 (9.3)  171 (0.5)  5 585 (15.4) |
| **Early life antibiotics exposure ^k^** | 36 013 (47.5) | 17 680 (44.7) | 18 333 (50.5) |
| **Maternal medical conditions:**  **Mood and anxiety disorders**  **Schizophrenia**  **Diabetes**  **Prenatal infections** | 5 779 (7.6)  73 (0.1)  2 163 (2.9)  26 693 (35.2) | 2 603 (6.6)  37 (0.1)  1 066 (2.7)  7 663 (19.4) | 3 176 (8.7)  36 (0.1)  1 097 (3.0)  19 030 (52.4) |
| **Prenatal medications use:**  **Antidepressants**  **Antipsychotics**  **Anticonvulsants**  **Cardiovascular medications** | 2 101 (2.8)  228 (0.3)  493 (0.7)  1 001 (1.3) | 969 (2.5)  101 (0.3)  209 (0.5)  487 (1.2) | 1 132 (3.1)  127 (0.4)  284 (0.8)  514 (1.4) |
| **Year of birth:**  **1998-2001**  **2002-2005**  **2006-2009**  **2010-2014** | 14 145 (18.6)  19 390 (25.6)  21 426 (28.2)  20 935 (27.6) | 7 329 (18.5)  10 472 (26.5)  11 285 (28.5)  10 491 (26.5) | 6 816 (18.8)  8 918 (24.6)  10 141 (27.9)  10 444 (28.8) |
| **Season of birth:**  **Winter**  **Spring**  **Summer**  **Fall** | 17 661 (23.3)  19 755 (26.0)  20 598 (27.1)  17 882 (23.6) | 9 258 (23.4)  10 127 (25.6)  10 727 (27.1)  9 465 (23.9) | 8 403 (23.1)  9 628 (26.5)  9 871 (27.2)  8 417 (23.2) |
| ^a^ Numbers (percentage). Percentages are calculated based on non-missing data  ^b^ Socio-Economic Factor Index (SEFI) was categorized with cut off points within one standard deviation from the mean into high, middle, low middle and low SES  ^c^ Defined as receiving income assistance for at least two months within 1 year before to 18 months after index date  ^d^ Missing data for 316 (0.4%) subjects  ^e^ Defined as the number of births following a multiple gestation pregnancy  ^f^ Defined as having birth weight below the 10^th^ percentile for the gestational age and sex. Missing data for 187 (0.2%) subjects  ^g^ Missing data for 36 810 (48.5%) subjects  ^h^ Missing data for 36 018 (47.5%) subjects  ^i^ Defined as having an infection code in physician claims only  ^j^ Defined as having a hospitalization with an infection code  ^k^ Defined as filling one or more antibiotic prescription during the first year of life identified in DPIN | | | |
